# Feasibility, Acceptability, and Preliminary Effectiveness of a Combined Digital Platform and Community Health Worker Intervention for Patients With Heart Failure: Pilot Randomized Controlled Trial

**DOI:** 10.2196/59948

**Published:** 2024-08-08

**Authors:** Jocelyn A Carter Carter, Natalia Swack, Eric Isselbacher, Karen Donelan, Anne Thorndike

**Affiliations:** 1 Division of General Internal Medicine Massachusetts General Hospital Harvard Medical School Boston, MA United States; 2 Corrigan Minehan Heart Center Massachusetts General Hospital Boston, MA United States; 3 Heller School for Social Policy and Management Brandeis University Waltham, MA United States

**Keywords:** heart failure, heart, cardiology, failure, clinical pilot trial, digital platform, home, digital health, remote monitoring, monitoring, home-based care, community health workers, social needs care, randomized controlled trial, controlled trials, feasibility, usability, acceptability, social needs

## Abstract

**Background:**

Heart failure (HF) is a burdensome condition and a leading cause of 30-day hospital readmissions in the United States. Clinical and social factors are key drivers of hospitalization. These 2 strategies, digital platforms and home-based social needs care, have shown preliminary effectiveness in improving adherence to clinical care plans and reducing acute care use in HF. Few studies, if any, have tested combining these 2 strategies in a single intervention.

**Objective:**

This study aims to perform a pilot randomized controlled trial assessing the acceptability, feasibility, and preliminary effectiveness of a 30-day digitally-enabled community health worker (CHW) intervention in HF.

**Methods:**

Adults hospitalized with a diagnosis of HF at an academic hospital were randomly assigned to receive digitally-enabled CHW care (intervention; digital platform +CHW) or CHW-enhanced usual care (control; CHW only) for 30 days after hospital discharge. Primary outcomes were feasibility (use of the platform) and acceptability (willingness to use the platform in the future). Secondary outcomes assessed preliminary effectiveness (30-day readmissions, emergency department visits, and missed clinic appointments).

**Results:**

A total of 56 participants were randomized (control: n=31; intervention: n=25) and 47 participants (control: n=28; intervention: n=19) completed all trial activities. Intervention participants who completed trial activities wore the digital sensor on 78% of study days with mean use of 11.4 (SD 4.6) hours/day, completed symptom questionnaires on 75% of study days, used the blood pressure monitor 1.1 (SD 0.19) times/day, and used the digital weight scale 1 (SD 0.13) time/day. Of intervention participants, 100% responded very or somewhat true to the statement “If I have access to the [platform] moving forward, I will use it.” Some (n=9, 47%) intervention participants indicated they required support to use the digital platform. A total of 19 (100%) intervention participants and 25 (89%) control participants had ≥5 CHW interactions during the 30-day study period. All intervention (n=19, 100%) and control (n=26, 93%) participants who completed trial activities indicated their CHW interactions were “very satisfying.” In the full sample (N=56), fewer participants in the intervention group were readmitted 30 days after hospital discharge compared to the control group (n=3, 12% vs n=8, 26%; *P*=.12). Both arms had similar rates of missed clinic appointments and emergency department visits.

**Conclusions:**

This pilot trial of a digitally-enabled CHW intervention for HF demonstrated feasibility, acceptability, and a clinically relevant reduction in 30-day readmissions among participants who received the intervention. Additional investigation is needed in a larger trial to determine the effect of this intervention on HF home management and clinical outcomes.

**Trial Registration:**

Clinicaltrials.gov NCT05130008; https://clinicaltrials.gov/study/NCT05130008

**International Registered Report Identifier (IRRID):**

RR2-10.2196/55687

## Introduction

Heart failure (HF) is a burdensome condition that affects over 64 million patients worldwide [1]. In the United States, total HF medical costs, mostly generated by inpatient hospitalizations [2], are estimated to increase from US $21 billion in 2012 to US $53 billion by 2030 [3]. HF is a leading cause of 30-day readmissions in the United States [4] and up to a quarter of these are preventable [5]. Key barriers to improving HF outcomes include the need for complex HF management at home reliant on tight adherence to clinical care plans (eg, medication, dietary, activity regimens) and unaddressed social needs often related to social determinants of health [6]. Despite important advances in 4-drug goal directed medical therapy and other evidence-based HF related treatments [7], few interventions have demonstrated impact in improving clinical outcomes in HF populations [8-11]. However, 2 strategies have generated encouraging findings for improving adherence to clinical care plans and reducing acute care use. The first is the use of digital platforms with remote monitoring, and the second is home-based care delivery from a navigator or community health worker (CHW).

Digital platforms have the potential to signal changes in biometrics to care teams (eg, body weight, blood pressure, changes in daily activity, and steps taken per day) while providing skill-based reinforcement of care plans and adherence to patients (eg, reminders and educational videos) [12-15]. While some digital studies have demonstrated benefit for clinically complex patients like those managing HF at home (eg, reducing days lost to unplanned readmissions, all-cause mortality, and increased activity) [16-18] results have generally been mixed [19-23]. Reasons for this include the lack of patient familiarity with digital platforms, suboptimal engagement with platform devices, and internet connectivity issues particularly in lower resourced, aging, or less technology inclined populations [24-28].

CHWs deliver home and community-based care as lay professionals acting as navigators in chronic disease populations [29,30]. CHW core competencies include motivational interviewing, psychosocial support, and goal setting. CHWs can strengthen connections to clinical teams by offering supportive health care coaching, identifying low and no cost resources related to food insecurity, transportation, rental or utility arrears, or even accompanying a patient to a clinical or social intake appointment [31-34]. Interventions that include CHW social needs care have demonstrated improvement in readmissions and medication adherence [35-37]. However, CHW care faces limitations of scale because it relies on mostly 1:1 care delivery requiring direct contact with patients for encounters [38-40]. Despite CHWs’ unique positioning to leverage real-time feedback generated by remote monitoring and enhance digital platform patient adoption [41], there are few examples in the literature of CHW integration with digital platform interventions [42,43].

We conducted a 30-day pilot randomized controlled trial (RCT) to determine the feasibility, acceptability, and preliminary effectiveness of a combined digital platform and CHW social needs care intervention compared to CHW social needs care alone for adults with HF and health-related social needs being discharged from the hospital.

## Methods

### Study Overview and Design

**Figure 1:**
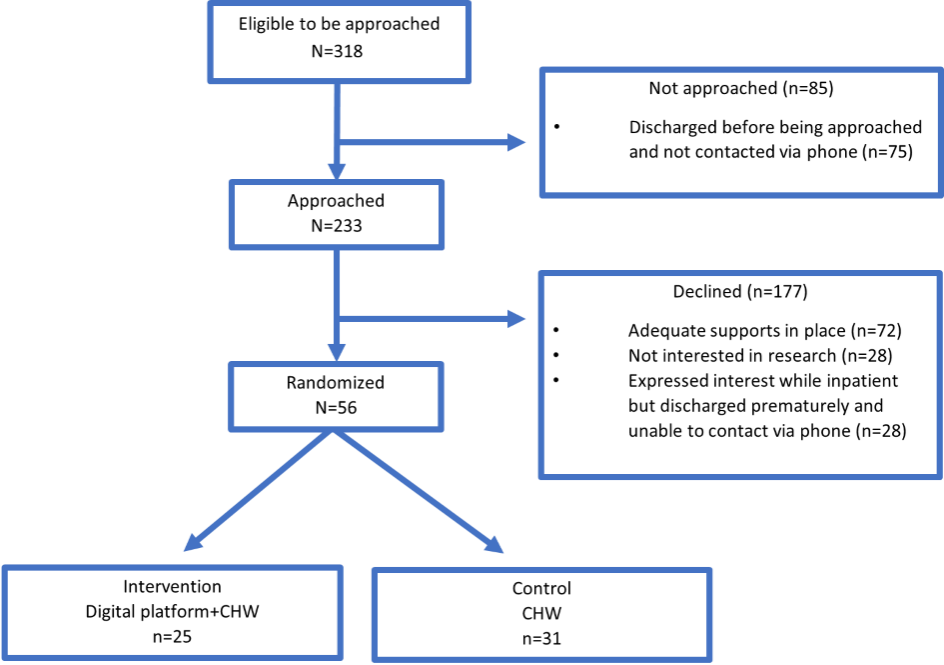
Enrollment study flow. CHW: community health worker.

This study was a RCT evaluating the intervention (digital platform + CHW + usual care) compared to the enhanced control (CHW + usual care) group over 30 days after hospital discharge. The trial methods have been previously described in detail [44]. Briefly, patients were screened for eligibility via the electronic medical record (EMR) on 8 inpatient study floors (6 internal medicine floors and 2 cardiology floors) at Massachusetts General Hospital (MGH), a 999-bed academic medical center in Massachusetts (Figure 1). Research staff verified eligibility, and then introduced the study to the patient. Study participants completed informed consent and enrollment questionnaires and then were randomized to the intervention or control arm for the 30-day study period. Participants were randomly allocated to either the intervention group or the control group using a REDCap (Research Electronic Data Capture; Vanderbilt University) computer-generated randomization sequence (blocks of 4). This process ensured the concealment of study arm allocation until after participants were consented and completed the enrollment questionnaire and mitigated risks associated with selection bias. Both intervention and control participants were contacted by an assigned CHW within 24 weekday hours of enrollment and received teaching via an American Heart Association sponsored patient education tool for HF. Intervention participants received the digital platform study equipment and were oriented to the use of all platform components by research staff prior to hospital discharge. All enrolled participants completed an exit questionnaire and interview via phone at the end of the 30-day intervention.

### Subject Eligibility and Recruitment Strategy

Eligibility criteria were established based on prior clinical trial and qualitative studies focused on care transitions from hospital to home [41,45-47]. Participant eligibility criteria included: being aged 18 years and older, living within a 30-mile radius of MGH, having a diagnosis of HF listed in the EMR problem list, having a history of ≥1 hospitalization within the previous 12 months, having a primary care or cardiologist clinician managing their HF, having cognitive ability to participate in the intervention, and being fluent in English. Ineligibility criteria included active alcohol or substance use disorder, long-term care facility residency, inability to provide consent, or active invoked health care proxy or prisoner status. Research staff attempted to enroll patients up to 3 times if they were unsure about participation or not available on initial approach. All participants were provided $250 at study completion as remuneration for participation.

### CHW Training and Supervision

CHW staff (n=2) were all trained in the core competencies of CHW care delivery for HF and other common diagnosis associated with hospital readmissions (eg, pneumonia, atrial fibrillation, pulmonary disease) [34]. CHW core competencies included motivational interviewing, goal setting, health care coaching, and psychosocial support grounded in a patient-centered and culturally competent CHW approach to socials needs care delivery. Supervision occurred through daily huddles (with a CHW staff supervisor) and weekly meetings with CHW staff supervisor and the principal investigator (JC). All clinical aspects of CHW care were supervised by the principal investigator. The care delivered in the intervention arm and control arms were administered by 2 different CHWs, respectively.

For the intervention arm, CHW staff received training on use of the digital platform, including how to assist patients with platform use and navigation [34]. Training fulfilled using participatory methods, case scenarios, and video clips for optimal teaching and implementation for the patient-facing app as well as the team dashboard. CHW staff were trained on how to interpret digital platform symptom assessments and biometric monitoring. Specifically, this included a machine learning–based daily score generated by the platform as well as alerts sent to the CHW team dashboard, indicating if participants were at or moving away from their clinical baseline in terms of symptoms (eg, shortness of breath and lower extremity swelling), biometrics (eg, body weight, blood pressure, and heart rate), and functionality (ie, steps taken daily). In conjunction with the dashboard, changes in the daily score, platform symptoms, and biometrics were translated to a color-coded schematic as a part of an algorithm to establish thresholds for outreach to clinical care teams, expedited in-home clinical evaluation, or expedited urgent or emergent care as previously described in a prior publication [44].

### Control Arm

Control arm participants were contacted routinely by telephone once a week or more by CHW staff to review medication adherence, nutrition, physical activity, symptoms, and clinic appointments and discuss any unmet social needs. If additional contacts were indicated based on participant needs or if participants preferred to receive resources via email or text contacts, CHW accommodated this consistently for all participants per protocol. A CHW staff member, with expertise in CHW core competencies (motivational interviewing, goal setting, behavior change, and psychosocial support) [30], identified resources to reduce gaps in care caused by unmet social needs and connected patients to clinical care teams for clinical questions. Daily huddles with the CHW supervisor occurred to discuss patient interactions and plans for goal achievement. CHW staff documented all participant encounters in the EMR. In addition, all CHW interactions were logged in a web-based research team REDCap database [48]. All social, behavioral, and clinical activities (clinical care team and community agency interactions, as well as time spent engaged in phone, in-person, and email modalities) were tracked. The patient’s clinical team members were copied on all EMR notes and contacted directly, when necessary, by the CHW or supervisory staff during the study. Control participants were encouraged to engage with CHW staff throughout the 30-day study interval.

### Intervention Arm

Prior to hospital discharge, intervention arm participants were introduced to the digital platform, a HF smartphone app (Android) that included a daily checklist and symptom questionnaire, educational HF videos, and a portal for CHW video visits. Launched in 2016, this HF digital platform [49] was designed for commercial use to help reduce 30-day readmissions in patients with HF by (1) leveraging artificial intelligence to minimize false alarms in biometric monitoring, (2) promoting early identification of decline in HF patients, and (3) encouraging digital and in-person communication between patients and care teams. Minor modifications to the smartphone app for CHW workflow integration were made for the purposes of this trial. In addition, participants were provided with a digital blood pressure monitor and a digital weight scale. A sensor attached to a lightweight arm band was worn on the nondominant arm and tracked basic biometric data (heart rate, oxygenation, and steps taken). A CHW staff member was trained to assist patients with technology set up and troubleshooting. Any unreconciled technical difficulties were addressed by research study staff and the platform vendor as needed. When CHW staff was notified by platform scores or alerts signaling that participants were moving away from their baseline, they discussed the patient’s findings with a research team member with clinical training (principal investigator and project manager). When indicated, CHW staff notified clinical team staff during weekday office hours within 2 hours of a biometric or other clinically related concern (ie, significant change in heart rate, blood pressure, body weight, or patient-reported symptoms). Participants were instructed to contact clinical care teams or seek urgent or emergent care as they would normally if they experienced symptomatic changes or other concerns outside weekday hours of operation.

Intervention participants were encouraged to connect with the CHW staff member, wear the digital sensor (tracking heart rate and steps taken daily) throughout the day or evening and measure blood pressure and weight daily using a digital blood pressure monitor and body weight scale (Multimedia Appendix 1). Similar to the control arm, a CHW staff member contacted participants routinely by telephone once week or more to review medication adherence, nutrition, physical activity, symptoms, clinic appointments, and to discuss any unmet social needs. If additional contacts were indicated based on participant needs or if participants preferred to receive resources via email or text contacts, CHW accommodated this consistently for all participants per protocol.

### Data Collection and Measures

All study participants completed an enrollment questionnaire focused on habits and patient experiences with home self-care [45,46]. Participants also completed exit questionnaires and exit interviews assessing their experience with CHW care or digitally-enabled CHW care in the control and intervention arms, respectively. For intervention participants, exit questionnaires included an acceptability questionnaire focused on the digital platform (limited to “very true,” “somewhat true,” and “not true” responses) and experience with the CHW (measured by a scale from “satisfied” to “neutral” to “not satisfied”). All questionnaires and exit interview prompts were initially pretested with 3 patients prior to making additional changes. All questionnaires (and the exit interview) were verbally administered by study staff. Basic demographics, insurance status, and major medical and psychiatric comorbidities were collected via chart review.

The primary outcomes were feasibility, acceptability, and preliminary effectiveness. Feasibility outcome measures included daily use rates of the biometric sensor (mean hours/day), the digital blood pressure monitor (mean times/day), the weight scale (mean times/day), and completion of the symptom questionnaire (mean times/day). The acceptability outcome measure was determined using patient responses to the truthfulness of a statement indicating willingness to use the intervention in the future (response options: very true, somewhat true, or not true). Preliminary effectiveness was measured by tracking 30-day clinical outcomes (hospital readmissions, emergency room visits, and missed primary care and cardiology appointments) occurred and these data were extracted from the electronic health record. All data was captured in REDCap.

### Statistical Analysis

Univariate analysis included demographic covariates of participants as well as intervention use frequencies, means, and SDs related to feasibility and acceptability outcomes. For the 30-day clinical outcomes of readmission, emergency department (ED), and missed primary care and specialty visits, we used the proportion with any readmissions, emergency visits, or missed clinic visits and compared between the 2 arms using Pearson c^2^ and Fisher exact tests. The number of readmissions, ED visits, or missed appointments was compared using Poisson models. Analyses of clinical outcomes were conducted using intention-to-treat principles.

### Ethical Considerations

Institutional review board approval was obtained from the Mass General Brigham Human Research Committee on September 22, 2020 (2018P002014). All enrolled participants provided written informed consent prior to this study. The original informed consent allows for secondary analysis without additional consent. All study data reported are deidentified. $50 remuneration was provided to participants at the time of enrollment and an additional $200 was provided after successful study completion. All methods were carried out in accordance with guidelines and regulations outlined by the Mass General Brigham Institutional Review Board.

## Results

Between September 2022 and June 2023, 56 eligible patients were enrolled and randomized (control: n=31; intervention: n=25). A total of 47 (84%) participants (control: n=28; intervention: n=19) completed all trial activities and were included in the final analysis (Figure 1). There were no significant differences in baseline characteristics between those randomized to the intervention and those randomized to the control arm (Table 1).

**Table 1:** Participant characteristics.

Participant characteristics			Control (n=31)		Intervention (n=25)
Sex (female), n (%)			17 (55)		11 (44)
Age (years), mean (SD)			69.4 (10.3)		61.6 (16.3)
**Race and ethnicity, n (%)**					
	Asian, non-Hispanic	1 (3)		2 (8)	
	Black, non-Hispanic	6 (19)		3 (12)	
	Hispanic or Latino	2 (7)		2 (8)	
	White, non-Hispanic	22 (71)		17 (68)	
	More than one race	0 (0)		1 (4)	
**Primary insurance, n (%)**					
	Medicare	19 (61)		12 (48)	
	Medicaid or MassHealth	1 (3)		1 (4)	
	Commercial or private	11 (36)		11 (44)	
	Other	0 (0)		1 (4)	
	Ejection fraction <40%	9 (29)		10 (40)	
**Highest level of education, n (%)**					
	High school or greater	13 (42)		6 (24)	
**Medical history, n (%)**					
	Hypertension	22 (71)		17 (68)	
	Coronary artery disease	14 (45)		6 (24)	
	Diabetes	13 (42)		9 (36)	
	Hyperlipidemia	12 (39)		9 (36)	
	Arrhythmia	11 (36)		12 (48)	
	Chronic kidney disease	11 (36)		8 (32)	
**Usage or needs, n (%)**					
	Hospitalizations in 12 months prior to enrollment	31 (100)		25 (100)	
**Technology perceptions, n (%)**					
	Indicated that they knew how to use a smartphone or app for health purposes	22 (71)		19 (76)	
	Indicated that a digital platform would be able to help them achieve their goals for managing their heart condition at home	19 (61)		16 (64)	

Intervention participants who completed trial activities (n=19) wore the digital sensor on 78% of study days with mean use of 11.4 (SD 4.6) hours/day. Intervention participants also completed daily symptom questionnaires on 75% of study days, used the blood pressure monitor 1.1 (SD 0.19) times/day, and used the digital weight scale 1 (SD 0.13) time/day. All intervention participants had ≥5 CHW interactions during the study interval and all intervention participants indicated that their CHW interactions were “very satisfying.” Of the control participants who completed trial activities (n=28), 25 (89%) had ≥5 CHW interactions and 26 (93%) indicated that their CHW interactions were “very satisfying.”

A total of 47 participants completed exit questionnaires. Of the intervention participants (n=19), all responded that the statement “I found that the different parts of the [digital platform] worked well together” was very true or somewhat true. All intervention participants indicated that the statement “If I have access to the [digital platform] moving forward, I will use it” was very true or somewhat true. Some of the intervention participants (n=9, 47%) indicated that the statement “I think I would need the support of a technical person” to use the digital platform was very true or somewhat true.

**Figure 2:**
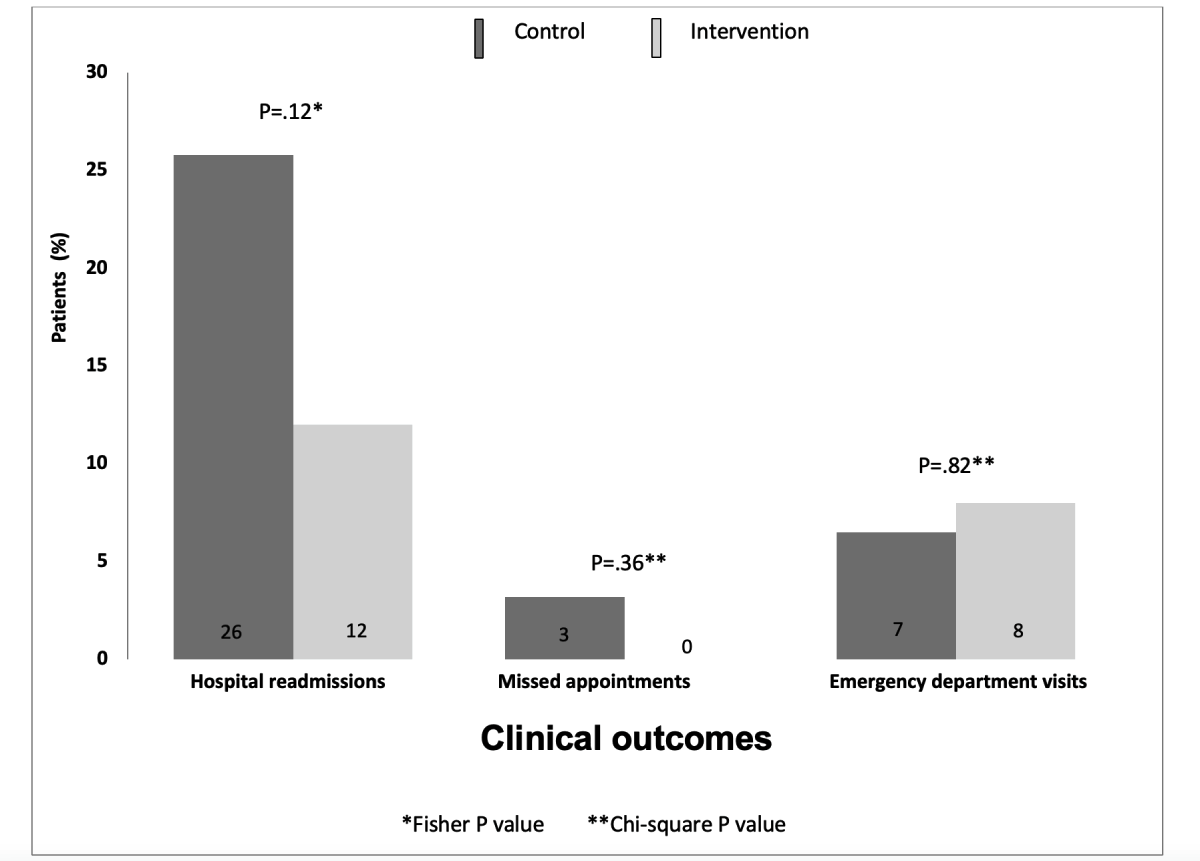
Postdischarge outcomes for digitally-enabled CHW versus CHW-enhanced usual care, September 2022-June 2023 (N=56). CHW: community health worker.

In an intention-to-treat analysis using the full sample (N=56), a lower proportion of participants in the intervention group compared to the control group was readmitted 30 days after hospital discharge (n=3, 12% vs n=8, 26%; *P*=.36; Figure 2). Both groups had similar proportions of participants with missed clinic appointments (n=0, 0% vs n=1, 3%; *P*=.22) and ED visits (n=2, 8% vs n=2, 7%; *P*=.82; Figure 2).

**Figure 3:**
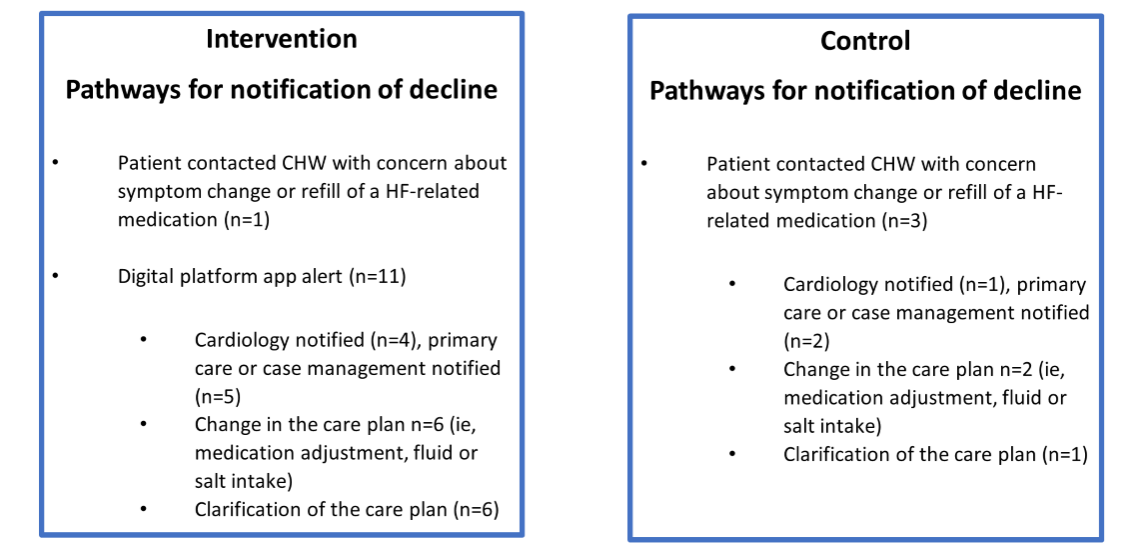
Examples of care team pathways for notification of patient decline. CHW: community health worker; HF: heart failure.

We identified several examples that resulted in additional CHW assessment, clinical team coordination, or care plan changes without resulting in acute care use or hospitalization (Figure 3). These examples, 12 in the intervention arm and 3 in the control arm, were triggered by patient symptoms or digital platform alerts relayed to the CHW staff. Subsequent involvement of the patient clinical care team members (intervention: n=9; control: n=3), medication changes (intervention: n=6; control: n=2), or clarification of the care plan (intervention: n=6; control: n=1) occurred on a case-by-case basis for intervention and control participants throughout the 30-day study period.

## Discussion

### Main Findings

In a pilot RCT, we found that an intervention combining a digital platform with CHW care for patients with HF was feasible and acceptable. Our findings also suggest that the intervention may have reduced 30-day hospital readmissions compared to CHW care alone. These results indicate that a digital platform designed for patients with HF and modified for use by trained CHW staff can be successfully implemented.

### Feasibility Findings

In the intervention group, most participants wore the sensor, used the digital BP and weight scale, and connected with the CHW staff throughout the study interval. Previous studies examining the feasibility of digital platforms interventions in HF identified similar levels of adoption and engagement [50,51]. We did not see differences in participant engagement or use associated with age that have been seen in some other studies [52]. This effect may be impacted by the inclusion of CHW staff whose training included digital platform troubleshooting and logistics resolution within a patient-centered and culturally competent framework.

### Acceptability Findings

Most intervention patients, despite limited prestudy digital health exposure, expressed willingness to use the intervention in the future [53]. A portion of intervention participants indicated they required assistance from someone to guide them through use of the digital platform. Other studies have highlighted the participant perceived technology-related difficulties and connectivity issues and barriers to platform adoption [54,55]. This underlies the potential impact of CHW pairings with the ability to contribute to navigation and engagement with the digital platform.

### Preliminary Effectiveness Findings

While this was a pilot trial with inadequate power to detect a statistically significant difference in clinical outcomes, the 13% absolute reduction in 30-day readmissions seen in the intervention arm as compared to the control was notable. A sustained 3% to 5% reduction in 30-day readmissions is generally considered ideal in scaled clinical settings [56]. This intervention was restricted to the 30-day period after hospital discharge; however, the reduction in 30-readmissions may signify the augmented value of combining a digital platform with CHW social needs care. The rates of ED visits and missed clinic appointments were not different between the intervention and control arms. This finding may be due to the similar effect of CHW care in both the intervention and control arms on reducing missed primary care [57] and ED visits [58]. Overall, these clinical findings suggest potential for health care savings and benefit to patients through the prevention of hospitalizations. Additional study will be needed to further define CHW and digital platform mechanisms of impact in this population.

### Limitations

There are limitations associated with this pilot trial. First, this trial was conducted using a small sample size, due to funding limitations. As a result, participants who dropped out of both arms after being enrolled impacted study momentum. This was largely due to patients disenrolling at or right after hospital discharge due to unexpected prior travel or other emerging commitments. While participant plans can change after any trial enrollment, we feel strongly that this occurred disproportionally in the peripandemic period. Second, all enrollment was at a single site academic urban hospital which limits generalizability. In addition, we were unable to enroll non–English-speaking participants because of limited funding for bilingual study materials and staff. In future studies, additional funding will allow us to expand the intervention to multiple sites and use a digital platform available in more than one language and supported by staff with corresponding language certifications. Furthermore, we hope for the digital platform to be available in additional languages so that non–English speaking patients can participate. Third, despite the use of validated self-reported measure of health care use in our exit questionnaire, we may not have identified all encounters of acute care use occurring outside the enrollment hospital system. However, all participants were within our hospital network system receiving most, if not all, of their care within designated accountable care organization coverage (meaning that all acute care use would be captured by our hospital EMR). Finally, healthy user bias may have occurred resulting in underrepresentation of patients with even higher rates of medical complexity.

### Conclusions

The findings of this trial demonstrated the feasibility, acceptability, and preliminary effectiveness of an innovative combined digital platform and CHW social needs care intervention. A larger-scale multisite randomized clinical trial is needed to determine the true effectiveness of this intervention with regard to clinical outcomes as well as which elements of the intervention (eg, interactions with CHWs, use of specific features of the digital platform) can offer the greatest value for patients characterized by specific demographic, clinical, and social domains.

## Appendix

Multimedia Appendix 1 Digital platform patient phone application screenshots.

Multimedia Appendix 2 CONSORT checklist.

## Abbreviations

CHW: community health worker
ED: emergency department
EMR: electronic medical record
HF: heart failure
MGH: Massachusetts General Hospital
RCT: randomized controlled trial
REDCap: Research Electronic Data Capture

## Data Availability

The data sets generated during and analyzed during this study are not publicly available due to the small size of this study but are available from the corresponding author on request.
