# Supplementary material for: Feasibility, Acceptability, and Preliminary Effectiveness of a Combined Digital Platform and Community Health Worker Intervention for Patients With Heart Failure: Pilot Randomized Controlled Trial
Source: JMIR Cardio. 2024 Aug 8;8:e59948. doi: 10.2196/59948 (PMC11342011; doi:10.2196/59948)
Supplement: Multimedia Appendix 1 [file cardio_v8i1e59948_app1.pdf]

Multimedia Appendix A

Digital remote monitoring device[1]

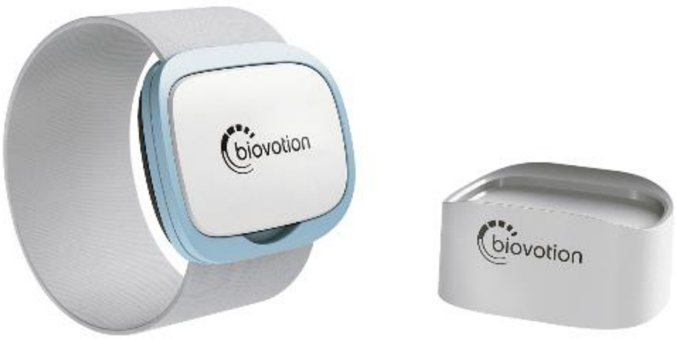

Continuous vital monitoring graphs[2]

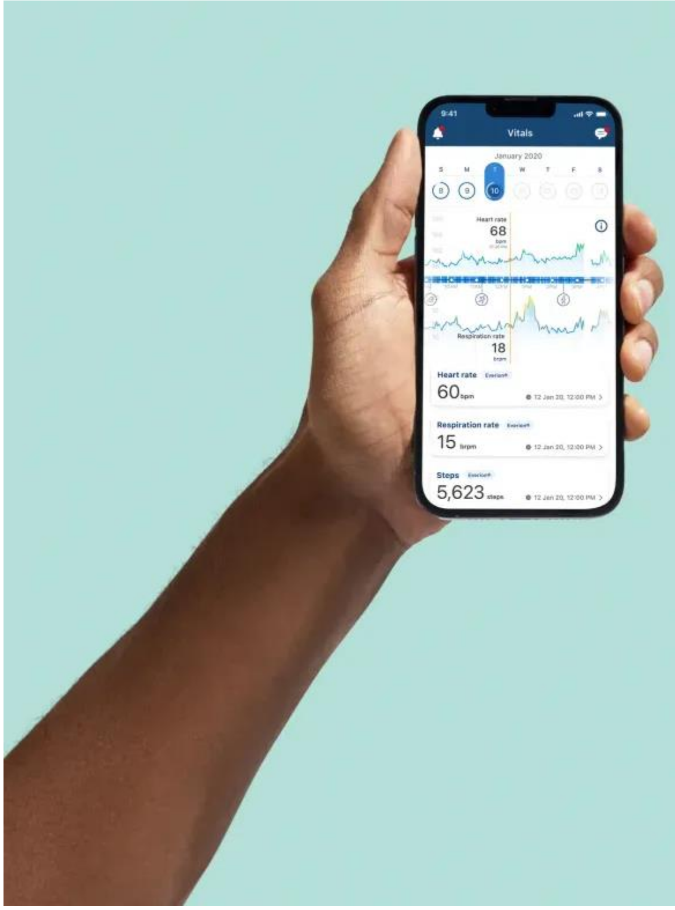

Mobile application home screen outlining daily tasks[3]

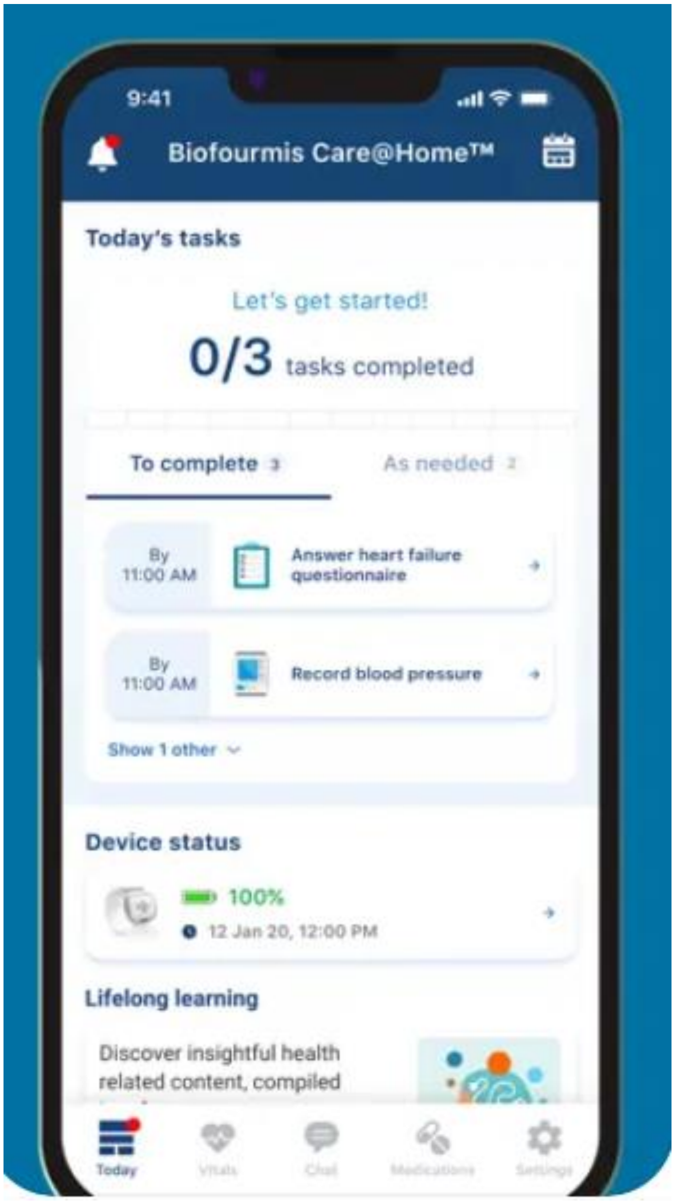

Mobile application vital measurement summary [3]

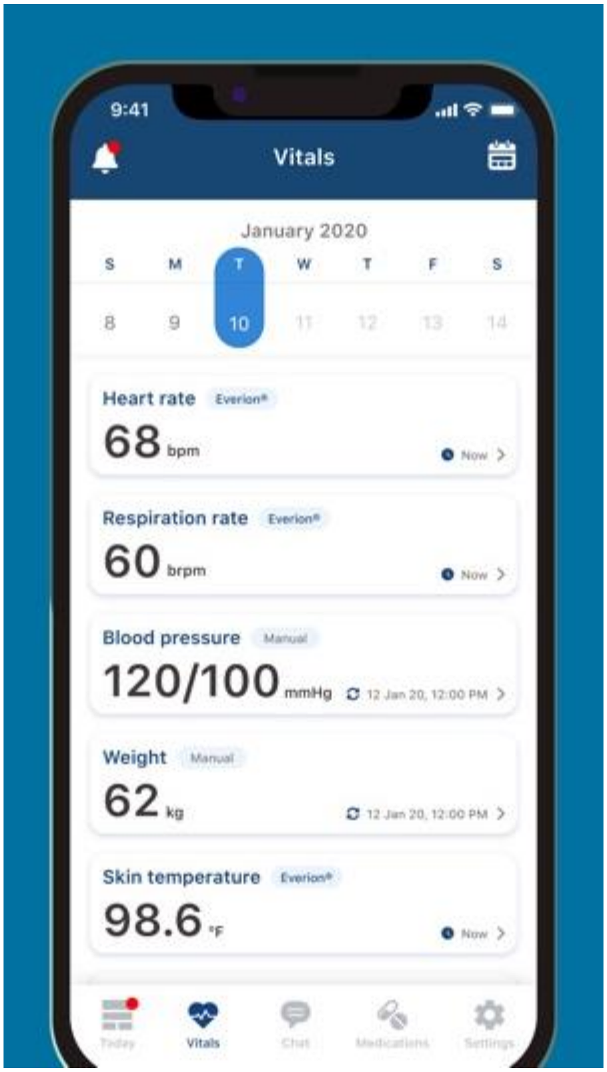

References

[1]https://www.medicaldevice-network.com/news/biofourmis-acquires-biovotion/

[2]https://hitconsultant.net/2024/02/15/wellspan-health-taps-biofourmis-to-expand-hospital-at-home-program/

[3]https://apps.apple.com/us/app/biofourmis-care-patient/id1544687811
